# A Molecular Neurobiological Approach to Understanding the Aetiology of Chronic Fatigue Syndrome (Myalgic Encephalomyelitis or Systemic Exertion Intolerance Disease) with Treatment Implications

**DOI:** 10.1007/s12035-018-0928-9

**Published:** 2018-02-06

**Authors:** Jean A. Monro, Basant K. Puri

**Affiliations:** 1 Breakspear Medical Group, Hemel Hempstead, England UK; 2 Department of Medicine, Imperial College London, Hammersmith Hospital, London, UK

**Keywords:** Chronic fatigue syndrome, Myalgic encephalomyelitis, Systemic exertion intolerance disease, Molecular neurobiology

## Abstract

Currently, a psychologically based model is widely held to be the basis for the aetiology and treatment of chronic fatigue syndrome (CFS)/myalgic encephalomyelitis (ME)/systemic exertion intolerance disease (SEID). However, an alternative, molecular neurobiological approach is possible and in this paper evidence demonstrating a biological aetiology for CFS/ME/SEID is adduced from a study of the history of the disease and a consideration of the role of the following in this disease: nitric oxide and peroxynitrite, oxidative and nitrosative stress, the blood–brain barrier and intestinal permeability, cytokines and infections, metabolism, structural and chemical brain changes, neurophysiological changes and calcium ion mobilisation. Evidence is also detailed for biologically based potential therapeutic options, including: nutritional supplementation, for example in order to downregulate the nitric oxide-peroxynitrite cycle to prevent its perpetuation; antiviral therapy; and monoclonal antibody treatment. It is concluded that there is strong evidence of a molecular neurobiological aetiology, and so it is suggested that biologically based therapeutic interventions should constitute a focus for future research into CFS/ME/SEID.

## Introduction

The disorder variously known inter alia as chronic fatigue syndrome (CFS), myalgic encephalomyelitis (ME) and systemic exertion intolerance disease (SEID) has a phenotype of unknown aetiology, whilst there is considerable controversy over the most appropriate treatment approach(es). In this in-depth review, we bring together the results of research into the molecular neurobiological mechanisms which underpin CFS/ME/SEID, thereby providing helping to inform an evidence-based approach to its treatment.

After briefly discussing the history and definition of this disorder, we consider a wide variety of molecular neurobiological factors and we then describe an evidence-based approach to the treatment of CFS/ME/SEID.

## History and Case Definition

### CFS/ME Clusters

From 1934 to 1990, there have been numerous documented clusters of outbreaks of CFS/ME [1–7]. These are summarised in Table 1.

**Table 1:** CFS/ME clusters from 1934 to 1990

• 1934 Los Angeles County Hospital: atypical poliomyelitis
• 1936 Fond Du Lac, Wisconsin—St. Agnes Convent: encephalitis
• 1937 Erstfeld, Switzerland: abortive poliomyelitis among 130 soldiers
• 1937 St. Gallen, Switzerland—Frohburg Hospital: abortive poliomyelitis among 28 staff members and patients
• 1939 Middlesex, England—Harefield Sanatorium: persistent myalgia
• 1939 Degersheim, Switzerland: abortive poliomyelitis among 73 soldiers
• 1945 Hospital of the University of Pennsylvania: epidemic pleurodynia
• 1946 Iceland: disease resembling poliomyelitis with the character of Akureyri disease
• 1948 Iceland, North Coast towns: epidemic simulating poliomyelitis
• 1949 Adelaide, South Australia: a disease resembling poliomyelitis
• 1950 Louisville, Kentucky—St. Joseph’s Infirmary: outbreak in nurses’ training school described as ‘epidemic neuromyasthenia’
• 1950 Upper State New York: outbreak resembling Iceland disease, simulating acute anterior poliomyelitis
• 1952 London, England—Middlesex Hospital Nurses’ Home: encephalomyelitis associated with poliomyelitis virus
• 1952 Copenhagen, Denmark: epidemic myositis
• 1952 Lakeland, Florida: epidemic neuromyasthenia
• 1953 Coventry and District, England: an illness resembling poliomyelitis observed in nurses
• 1953 Rockville, Maryland—Chestnut Lodge Hospital: poliomyelitis-like epidemic neuromyasthenia among student nurses
• 1953 Jutland, Denmark: epidemic encephalitis with vertigo
• 1954 Tallahassee, Florida: ‘a new clinical entity?’
• 1954 Seward, Alaska: benign ME (Iceland disease)
• 1954 Berlin—British army: further outbreak of a disease resembling poliomyelitis
• 1954 Liverpool, England: outbreak among medical and nursing staff in a local hospital
• 1955 Dalston, Cumbria, England: epidemic and sporadic outbreak of an unusual disease
• 1955 London, England—Royal Free Hospital: benign ME
• 1955 Perth, Australia: virus epidemic in waves
• 1955 Gilfac Goch, Wales: benign ME
• 1955 Durban City, South Africa—Addington Hospital: outbreak among nurses of ‘Durban mystery disease’
• 1955 Segbwema, Sierra Leone: outbreak of encephalomyelitis
• 1955 Patreksfjorour and Porshofn, Iceland: unusual response to polio vaccine
• 1955 Northwest London, England—nurses’ residential home: acute infective encephalomyelitis simulating poliomyelitis
• 1956 Ridgefield, Connecticut: epidemic neuromyasthenia
• 1956 Punta Gorda, Florida: outbreak of epidemic neuromyasthenia
• 1956 Newton-le-Willows, Lancashire, England: lymphocytic meningoencephalitis with myalgia and rash
• 1956 Pittsfield and Williamstown, Massachusetts: benign ME
• 1956 Coventry, England: epidemic malaise, benign ME
• 1957 Brighton, South Australia: Cocksackie echo virus meningitis, epidemic ME
• 1958 Athens, Greece—nurses’ school: outbreak of benign ME with periostitis and arthopathy noted
• 1958 Southwest London, England: reports of sporadic cases of ME
• 1959 Newcastle Upon Tyne, England: outbreak of benign ME
• 1961 Basel, Switzerland: sporadic cases of benign ME
• 1961 New York State: outbreak of epidemic neuromyasthenia in a convent
• 1964 Northwest London, England: epidemic malaise, epidemic neuromyasthenia
• 1964 Franklin, Kentucky: outbreak of neuromyasthenia in a factory
• 1965 Galveston, Texas: epidemic neuromyasthenia variant
• 1967 Edinburgh, Scotland: sporadic cases resembling benign ME
• 1968 Fraidek, Lebanon: benign ME
• 1969 Brooklyn, New York—State University of New York Downstate Medical Center: epidemic neuromyasthenia, unidentified symptom complex
• 1970 Lackland Air Force Base, Texas: epidemic neuromyasthenia
• 1970 London, England—Great Ormond Street Hospital for Children: outbreak of neuromyasthenia among nurses
• 1975 Sacramento, California—Mercy San Juan Hospital: infectious venulitis, epidemic phelobodynia
• 1976 Southwest Ireland: epidemic neuromyasthenia, benign ME
• 1977 Dallas—Fort Worth, Texas: epidemic neuromyasthenia
• 1979 Southampton, England: ME
• 1980 West Kilbridge, Ayrshire, Scotland: epidemic ME
• 1980 Helensburgh, Scotland: Cocksackie B outbreak in a private practice
• 1980 San Francisco, California: epidemic persistent flu-like illness
• 1981 Stirlingshire, Scotland: sporadic ME
• 1981 Gunnedah, NSW, Australia: outbreak linked with pesticides
• 1983 Los Angeles, California: initial cases of an unknown, chronic symptom complex involving profound ‘fatigue’
• 1984 West Otago and Tapanui, Dunedin and Hamilton, New Zealand: ME
• 1984 Lake Tahoe–Truckee area of California/Nevada: start of a year-long epidemic involving > 160 cases of chronic illness eventually characterised as CFS
• 1984 Yerington, Nevada: epidemic of about 100 cases on a Native American reservation, eventually characterised as CFS
• 1984 Chapel Hill, North Carolina: epidemic among members of North Carolina Symphony Orchestra, eventually characterised as CFS
• 1984 Montreal, Quebec—Ontario, Canada: > 500 cases documented and eventually characterised as CFS
• 1985 Lyndonville, New York: epidemic among children eventually characterised as CFS
• 1986 Placerville, California: epidemic eventually characterised as CFS
• 1986 Sonora, California: epidemic of 35 children and adults, mostly associated with Columbia Community College, eventually characterised as CFS
• 1988 Narrabeen, NSW, Australia: outbreak reported
• 1989 Roseville, California: outbreak of 11 cases of CFS among staff at Rosedale Hospital
• 1990 Elk Grove, California: outbreak among teachers and students at a high school
• 1990 Mohave Valley Region, Arizona: > 100 people ill with a ‘multi-system stealth virus infection with encephalopathy’

### Diagnostic Criteria

Here, we consider several sets of diagnostic criteria and case definitions which have been published since the early 1990s.

In 1991, the Oxford criteria for CFS of Michael Sharpe (based at the University of Oxford) and colleagues were published [8]. These require the presence of severe disabling fatigue of at least a 6-month duration affecting both physical and mental functioning, and which is present for more than half the time.

The revised Centers for Disease Control and Prevention (CDC) criteria published in the *Annals of Internal Medicine* in December 1994 [9], and including Sharpe as a co-author, have been widely used in academic research into CFS/ME. After excluding any other cause for chronic fatigue, they require self-reported persistent or relapsing fatigue for at least six consecutive months and the concurrent presence, for over 6 months, of at least four of the following sets of symptoms: impaired memory or concentration; sore throat; tender cervical or axillary lymph nodes; myalgia; multi-joint pain; new headaches; unrefreshing sleep; post-exertion malaise. Interestingly, whereas the revised CDC criteria count major depressive disorder as an exclusion criterion, this is not the case with the earlier Oxford criteria.

In 2003, the Canadian consensus criteria of Carruthers and colleagues were published and required meeting specific criteria for fatigue, post-exertional malaise and/or fatigue, sleep dysfunction and pain; the presence of at least two neurological/cognitive manifestations; the presence of at least one symptom from two of the categories of autonomic, neuroendocrine and immune manifestations; and an illness duration of at least 6 months (3 months for children) [10, 11]. A revised set of criteria were published by Jason and colleagues in 2010, which included a questionnaire to assess core symptoms and which specified explicit rules for determining whether critical symptoms met the following revised criteria: persistent or recurring chronic fatigue over the previous 6 months which is not lifelong and which results in substantial reductions in previous levels of occupational, educational, social and personal activities; post-exertional malaise and/or post-exertional fatigue; unrefreshing sleep or disturbance of sleep quantity or rhythm disturbance; pain or discomfort; at least two neurological/cognitive manifestations; at least one symptom from two of the three categories autonomic, neuroendocrine and immune; and the absence of exclusionary conditions [12].

An international consensus panel of clinicians, researchers, teaching faculty and an independent patient advocate from Canada, Belgium, the USA, the UK, Ireland, Australia, New Zealand, Norway, Italy, South Korea, Chile, Japan and Latvia developed a new set of consensus criteria, which were published in 2011 [13]. They pointed out the overlap between the CDC criteria and depressive symptoms, and suggested that there was no need for the 6-month temporal criterion of the Canadian consensus criteria. They also much preferred the name ME, ‘[i]n view of … research and clinical experience that strongly point to widespread inflammation and multisystemic neuropathology’ rather than a name which included the words ‘chronic fatigue’, pointing out that diagnoses such as cancer and multiple sclerosis (MS) do not have these two words appended to their names [13]. The ME International Consensus Criteria require that a patient meet the criteria for post-exertional neuroimmune exhaustion, at least one symptom from three neurological impairment categories, at least one symptom from three immune/gastrointestinal/genitourinary impairment categories and at least one symptom from energy metabolism/transport impairments [13].

In 2015, the US Institute of Medicine published their report on this disorder, which they suggested be renamed SEID; they argued that ME does not describe the illness accurately while the name CFS ‘can result in trivialization and stigmatization for patients afflicted with this illness’; it was stressed that SEID is a medical illness rather than a psychiatric or psychological one [14]. The diagnostic criteria include: a substantial reduction or impairment in the ability to engage in pre-illness levels of occupational, educational, social or personal activities that persist for more than 6 months, is accompanied by fatigue, is of new or definite onset (not lifelong), is not the result of ongoing excessive exertion and is not substantially alleviated by rest; post-exertional malaise; unrefreshing sleep; and cognitive impairment and/or orthostatic intolerance [14].

## Nitric Oxide and Peroxynitrite

### The NO/ONOO^−^ Cycle

**Fig. 1:**
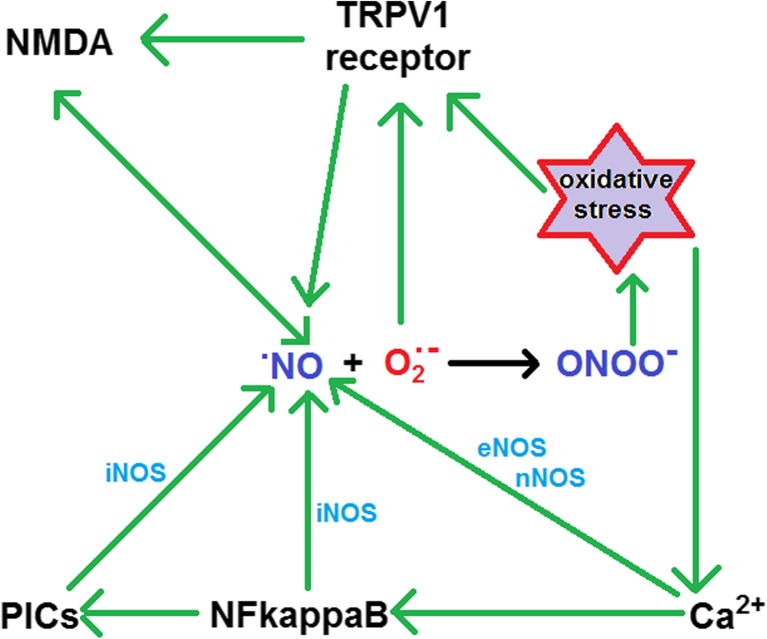
Key aspects of the nitric oxide–peroxynitrite cycle. See text for details (partly based upon figure 1.2 in reference [15])

Pall has described a vicious cycle which is initiated by nitric oxide (NO; official name—nitrogen monoxide) and peroxynitrite (ONOO^−^; official name—oxoperoxonitrate (1−)) [15, 16]. Although Pall refers to this as the NO/ONOO^−^ cycle, it could also be referred to as the ^•^NO/ONOO^−^ cycle since the nitrogen in NO has an unpaired electron in the π*(2p) antibonding orbital, meaning that the nitric oxide is a free radical. While the whole cycle needs to be considered in any individual, interlocking cyclic components of it can be scrutinised. Some important aspects of such components of the ^•^NO/ONOO^−^ cycle are now described and are illustrated in Fig. 1.

In terms of energy metabolism, it is noted first that excessive nitric oxide produces more superoxide and then a depletion of adenosine triphosphate (ATP). This leads to activation of *N*-methyl-d-aspartate (NMDA) through its receptors. This can result in heightened intracellular calcium, which in turn will trigger increased activity of endothelial nitric oxide synthase (eNOS) and of neuronal nitric oxide synthase (nNOS) and perpetuation of increased nitric oxide. Furthermore, superoxide can activate transient receptor potential (TRP) channels and NMDA. These work principally through vanilloid TRPV1 receptors, which are activated in multiple chemical sensitivity (MCS) [17–20]. When TRP receptors, active NMDA receptors and calcium are increased, this results in voltage-gated calcium channels being activated, and Pall has implicated this mechanism in food and chemical sensitivity [17–20].

Another cyclic component of the overall ^•^NO/ONOO^−^ cycle involves the reaction with nitric oxide with superoxide to form peroxynitrite, as follows:

$$ {}^{\bullet}\mathrm{NO}+{{\mathrm{O}}_2}^{\bullet -}\to {\mathrm{O}\mathrm{NO}\mathrm{O}}^{-} $$

Thus, elevated nitric oxide leads to increased superoxide and peroxynitrite. This leads to oxidative stress and upregulation of the protein complex nuclear factor kappa-light-chain enhancer of activated B cells (NFκB), with many pro-inflammatory cytokines (PICs) being induced. These in turn increase the activity of inducible nitric oxide synthase (iNOS), which increases nitric oxide. Oxidative stress itself can also lead to increased calcium, which triggers nitric oxide production, while NFκB and PICs can be provoked by infections [21]. In addition to superoxide provoking a depletion of ATP (see above), peroxynitrite can also do so [15, 16].

Peroxynitrite oxidises the electron carrier tetrahydrobiopterin (BH4), which is derived from the cofactor biopterin. BH4 acts as a NOS cofactor as well as playing a role in the degradation of aromatic amino acids and the biosynthesis of catecholamine and serotonin/melatonin [22]. For example, in the hydroxylation of phenylalanine to tyrosine (catalysed by phenylalanine hydroxylase), BH4 is converted into quinonoid dihydrobiopterin; the latter is reduced, by nicotinamide adenine dinucleotide (NADH), back into BH4 (catalysed by dihydropteridine reductase).

### Changes in CFS/ME/SEID

Pall has adduced evidence that various components of his NO/ONOO^−^ cycle are abnormal in CFS/ME/SEID [21, 22]. First, the following stressors, which have been implicated in the initiation of CFS/ME/SEID, can lead to increased nitric oxide levels: infection by viruses, bacteria and protozoa; carbon monoxide exposure; ciguatoxin poisoning; physical trauma; psychological stress; and exposure to ionising radiation [22]. Second, many studies have reported that CFS/ME/SEID is associated with elevated levels of markers of oxidative stress, nitric oxide levels and PICs (reviewed by reference [22]).

Chronic activation of NFκB is hypothesised to occur in CFS/ME/SEID (see Fig. 1); in 2011, Hoeck and Pall suggested that vitamin D_3_ (1,25(OH)_2_D_3_) supplementation may be helpful in this disease owing to the ability of active vitamin D_3_ to repress activation of NFκB, via binding to vitamin D receptor [23]. To date, there are no published trial data testing this suggestion. However, 3 years later, Witham and colleagues independently reported that in CFS/ME patients, the level of vitamin D_3_ is inversely correlated with markers of inflammation, oxidative stress and cardiovascular risk [24]. NFκB activation is likely to occur in response to ionising radiation, via free radical and pro-oxidant molecules, and Pall has hypothesised that this may constitute part of the mechanism for symptoms of post-radiation syndrome, which is a CFS/ME/SEID-like disorder [25].

In 2003, Smirnova and Pall reported that the serum protein carbonyl levels are elevated in CFS patients compared with controls, while there is no significant difference in total protein levels [26]. Since protein carbonyl levels index protein oxidation, this finding is consistent with increased oxidative stress in CFS/ME and therefore offers support for Pall’s NO/ONOO^−^ cycle model (see Fig. 1).

Both CFS/ME/SEID and cardiac failure are associated with fatigue [27]. In 2013, Pall published extensive evidence, including 34 mechanisms, pointing to the NO/ONOO^−^ cycle as being central to the cause of cardiac failure [28]. There is also evidence of an association between CFS/ME/SEID and multiple chemical sensitivity (MCS) [29–31]. Pall has suggested that MCS may be a response to the index chemicals causing increased NMDA activity (see Fig. 1) [17, 19]. Again, in 2013, Pall showed how volatile chemicals may act as toxicants via both transient receptor potential ankyrin 1 (TRPA1) and TRPV1 receptors; the latter are part of the NO/ONOO^−^ cycle (see Fig. 1) [18, 32, 33].

## Oxidative and Nitrosative Stress

Fukuda and colleagues recently showed that, at rest, measures of oxidative stress were higher, and measures of biological antioxidant potential lower, in CFS patients than in healthy volunteers [34]. These may well adversely impact on lipoprotein-based cellular signalling; Morris and colleagues have pointed out that oxidative and nitrosative stress (O&NS) affects the following lipid-based signalling mechanisms: the post-translational *S*-palmitoylation modification; the functions of omega-3 polyunsaturated fatty acids (PUFAs); and the functions of membrane/lipid rafts [35].

A recent study of the use of nutraceuticals with actions against inflammatory, O&NS in 76 CFS/ME patients was carried out by Maes and Leunis [36]. At baseline, the patients had abnormal autoimmune responses. Improved clinical outcome was associated with a reduction in autoimmune responses to oxidative specific epitopes, such as malondialdehye (MDA) and phosphatidyl inositol, rather than to a reduction in nitroso-adducts such as nitroso-arginine, nitroso-cysteinyl and nitroso-tryptophan [36].

## Blood–Brain Barrier Damage and Intestinal Permeability

When the blood–brain barrier is damaged, circulating antibodies that cross-react with neurological tissues can infiltrate the brain and nervous tissue, with the potential destruction of neurological tissues. The cycle of neuroautoimmunity can begin with a breach of the gastrointestinal and/or blood–brain barriers. It is also important to bear in mind that there exist gaps in the blood–brain barrier to allow hypothalamic-hormonal control via biofeedback; therefore, the (neuro-)lymphatic/glymphatic drainage is important for brain health and possibly for CFS/ME/SEID [37–41].

Cross and colleagues have carried out murine experiments on acute experimental autoimmune encephalomyelitis (which serves as an animal model for human multiple sclerosis) which show that nitrotyrosine is found in the CNS after exposure to peroxynitrite [42]. The formation of nitrotyrosine under physiological conditions is indicative of peroxynitrite damage of proteins (including the amino acid tyrosine) [42, 43]. The molecular mechanism is likely to involve the formation of the intermediate nitrosoperoxycarbonate (O=NOOCO_2_^−^) by the following reaction:

$$ {\mathrm{ONOO}}^{-}+{\mathrm{CO}}_2\to {{\mathrm{ONOO}\mathrm{CO}}_2}^{-} $$

followed by homolytic fission of the nitrosoperoxycarbonate to two reactive radicals:

$$ {{\mathrm{ONOOCO}}_2}^{-}\to {}^{\bullet }{\mathrm{NO}}_2+{{\mathrm{CO}}_3}^{\bullet -} $$

Following oxidation of tyrosine by the second of these two radicals (the carbonate radical, CO_3_^•−^), the product, tyr-O^•^, then combines with the first radical, ^•^NO_2_, to form nitrotyrosine [43]. Since nitrotyrosine is found within the CNS following peripheral exposure to peroxynitrite, it seems reasonable to conclude that peroxynitrite damages the blood–brain barrier; given the evidence favouring increased peroxynitrite activity in CFS/ME/SEID, blood–brain barrier damage in this disorder may therefore be expected [44].

As alluded to earlier, viral infections and PICs are associated with increased ^•^NO levels, often as a result of increased iNOS expression [45–47], which in turn leads to increased peroxynitrite levels. Again, by the above mechanism, this may lead to blood–brain barrier damage [42, 44]. Evidence of the association of infections and PICs, on the one hand, with CFS/ME/SEID, on the other hand, is given in later sections of this paper.

Staines and colleagues have hypothesised that immunopathology of the cerebrospinal perivascular compartment may occur in CFS/ME/SEID, with the particular involvement of the vasoactive neuropeptides pituitary adenylate cyclase-activating polypeptide (PACAP) and vasoactive intestinal peptide (VIP) [48]. Breaching of these barriers may result in the damaging effects of Th1 and Th17 lymphocytes, as well as antibodies that can target and damage neurones and tissues [49, 50]. Furthermore, elevated cerebrospinal fluid (CSF) levels of the PIC tumour necrosis factor-alpha (TNF-α) have been reported in CFS patients compared with non-CFS controls [51].

Environmental exposures of sufficient magnitude, including gut dysbiosis, may also trigger opening of tight junctions in the gastrointestinal tract, leading to the entry into the circulation of lipopolysaccharide (LPS) molecules, as well as undigested molecules and bacterial toxins; similarity between some gastrointestinal endothelial tissue proteins and certain proteins of the blood–brain barrier may lead to ‘leaky brain’ [52, 53]. In a rigorously controlled study of 50 CFS/ME patients compared with 50 matched healthy controls, CFS/ME was indeed found to be associated with gut dysbiosis [54]. The importance of this phenomenon in CFS/ME/SEID needs further study.

## Cytokines and Infections

The finding of increased CSF TNF-α in CFS has been mentioned above [51]. Several other studies have confirmed that CFS/ME/SEID is associated with increased levels of cytokines, particularly those which are pro-inflammatory. Four further recent examples are described here.

Using a high-throughput 51-multiplex array, Montoya and colleagues studied serum cytokines in 192 ME/CFS patients and 392 healthy controls in the important Stanford Myalgic Encephalomyelitis Initiative [55]. The following cytokines showed a significant elevation which correlated with disease severity: CCL11 (Eotaxin-1), CXCL1 (GROα), CXCL10 (IP-10), IFN-γ, IL-4, IL-5, IL-7, IL-12p70, IL-13, IL-17F, leptin, G-CSF, GM-CSF, LIF, NGF, SCF and TGF-α; of these, 13 are PICs and are likely to contribute to symptoms [55].

Using a 51-multiplex array, Hornig and colleagues studied CSF cytokines in 32 patients and 19 matched controls; their results pointed to a disturbance in IL-1 signalling [56]. In an earlier study, Horning and fellow workers also found evidence of cytokine activation and dissociation of inter-cytokine regulatory networks early in the course of CFS/ME, with illness duration being better correlated with cytokine changes than illness severity [57].

A study by Milrad and colleagues showed that poor sleep quality in CFS/ME patients is associated with higher levels of the PICs TNF-α, IL-1β and IL-6 [58].

These studies suggest that CFS/ME/SEID has a dynamic immunopathology with CNS immune activation and a shift to the Th2 pattern seen in autoimmunity [56, 57]. Furthermore, neuroinflammation, together with O&NS and neural sensitivity, can be formulated into a robust model of the aetiopathology of CFS/ME/SEID involving sustained glial activation [59].

As mentioned earlier, increased PIC levels are associated with infections. It is therefore noteworthy that Underhill has suggested that CFS/ME is an infectious disease, on the basis of clinical, epidemiological and immunological evidence [60]. The following evidence particularly supports the role of Epstein–Barr virus (EBV) or human herpesvirus-4 (HHV-4), which can cause infectious mononucleosis, in this regard.

At 6 months, 1 year and 2 years after infection, of 301 adolescents, aged between 12 and 18 years, with infectious mononucleosis, 13, 7 and 4%, respectively, met CFS criteria in a study by Katz and colleagues [61]. While CFS/ME/SEID patients and controls have been found to have similar IgG antibody response patterns to EBV, patients show increased IgG reactivity to an EBNA-6 repeat sequence and also to EBNA-6 protein; it may be that homologous sequences of human proteins containing this sequence could give rise to antigenic mimicry [62].

It is also interesting to note that there is evidence that CFS/ME/SEID patients suffer from sensitised fatigue pathways [63], as this may be related to the spread of reactivated EBV to ectopic lymphoid aggregates [64].

Besides EBV, Montoya and colleagues have suggested that HHV-6 may also be an infectious trigger for CFS/ME [65]. Importantly, Pantry and colleagues have shown that chromosomal integrated HHV-6 can be associated with persistent infection with exogenous HHV-6, which in turn may be associated with neurological symptomology [66].

## Metabolism

There is now clear evidence of metabolic dysfunction in CFS/ME/SEID. As mentioned above, CFS/ME/SEID is associated with the presence of chronic O&NS, low-grade inflammation and impairment of the production of heat shock proteins [67]. Naviaux and colleagues studied 45 patients and 39 age- and sex-matched controls using targeted, broad-spectrum metabolomics and found evidence that, in this disorder, 20 examined metabolic pathways were abnormal, including those involved in mitochondrial and peroxisomal metabolism and those involved in the metabolism of branched-chain amino acids, cholesterol, phospholipids, purines, pyrroline-5-carboxylate, riboflavin, sphingolipid, as well as microbiome metabolism [68]. In a more recent metabolic profiling study, abnormalities in purine, pyrimidine and amino acid metabolic pathways were found (including adenosine diphosphate and ATP) as well as in fatty acid and lipid metabolism [69]. There is also evidence of impaired pyruvate dehydrogenase function [70], which is consistent with a shift from oxidative phosphorylation to the production of lactic acid in this disease.

**Fig. 2:**
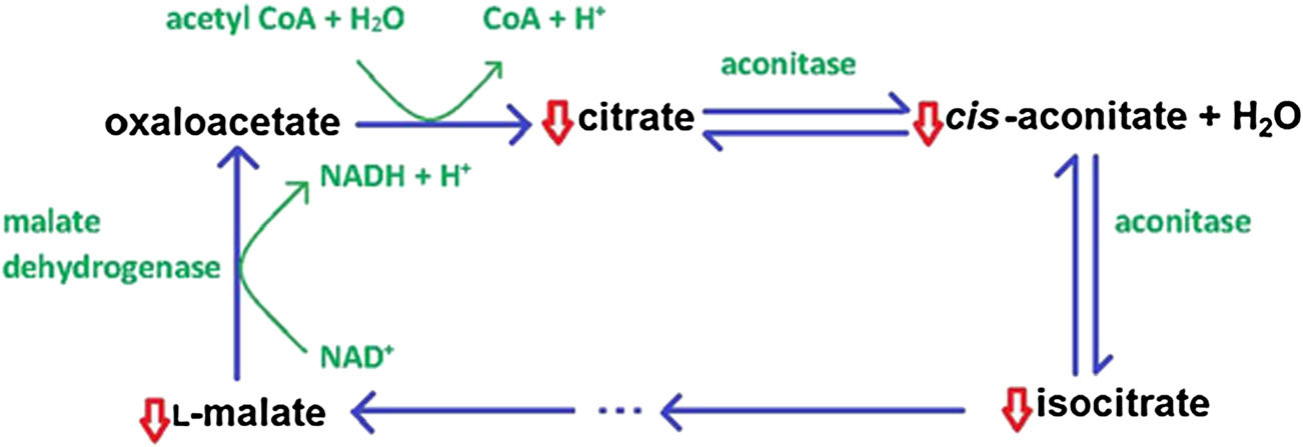
Metabolic changes in Kreb’s cycle reported in CFS/ME/SEID (based on data in reference [71])

**Fig. 3:**
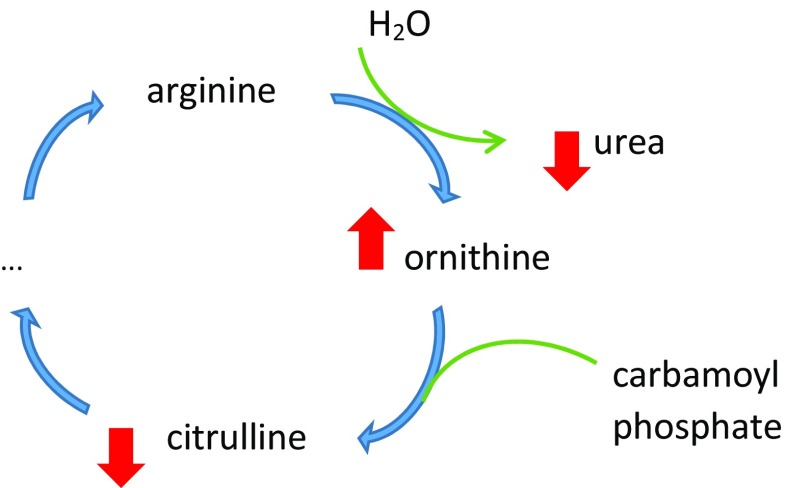
Metabolic changes in the urea cycle reported in CFS/ME/SEID (based on data in reference [71])

In their comprehensive metabolomic analyses of plasma samples from 46 CFS patients and 47 age- and sex-matched controls, Yamano and fellow co-workers reported group differences in both the Kreb’s (tricarboxylic or citric acid) cycle, with lower citrate, lower *cis*-aconitate, lower isocitrate and lower malate in the patient group (illustrated in Fig. 2), and in the urea cycle, with the patient group showing higher ornithine, lower citrulline and lower urea levels (see Fig. 3) [71].

## Brain Changes

One of the largest voxel-based morphometry brain MRI cross-sectional studies at a 3-T magnetic field strength was that carried out by Puri and colleagues, which compared 26 CFS/ME/SEID patients with 26 age- and sex-matched controls [72]. The patient group had reduced grey matter in the following three regions. First, the occipital lobes, including both poles, the superior division of the left lateral cortex and the supracalcrine cortex also on the left side of the brain. Second, the angular gyrus of the right hemisphere. Third, the posterior division of the left parahippocampal gyrus. Reduced white matter was found in the patient group in the left occipital lobe [72]. These particular findings are consistent with the symptom of impaired memory which is well described in CFS/ME/SEID, and they also point to the possibility of visual processing abnormalities and the possibility of discrepancies existing between intentions and corresponding actions [72]. This has been described as just one of several structural neuroimaging studies which have shown that CFS/ME/SEID is associated with significant neuroanatomical changes.

Furthermore, as Shan and colleagues found in their longitudinal MRI study of 15 patients and 10 controls, who were scanned (at 1.5 T) at baseline and 6-year follow-up, significant neuroanatomical changes occur over time [73]. In the case of this study, there was a decrease in white matter in the left inferior fronto-occipital fasciculus, which was not seen in the control group [73]. It should be noted, however, that not all recent longitudinal MRI studies have shown evidence of changes. Notably, the longitudinal study of Perrin and colleagues proved negative [74].

The chemistry of the brain has also been studied in this disorder, using magnetic resonance spectroscopy (MRS). The first systematic proton-MRS study of CFS/ME patients compared with age- and sex-matched controls was that of Puri and colleagues, who in 2002 reported that CFS was associated with a relative increase of choline-containing compounds in the occipital cortex [75]. The following year, Chaudhuri and colleagues similarly systematically studied the left basal ganglia of CFS/ME patients and age- and sex-matched controls using proton-MRS; they also found a relative increase of choline-containing compounds in this part of the brain [76]. These MRS findings may point to a problem with phospholipid metabolism, increased membrane turnover owing to gliosis or changes in intra-membrane signalling [75, 76]. They may also be consistent with Perrin’s much earlier prediction of increased central levels of acetylcholine [77, 78].

Thus, there is good evidence of the existence of neuroanatomical and neurochemical changes in CFS/ME/SEID. This is also ample evidence of neurophysiological changes, which are discussed in the next section.

## Neurophysiological Changes

Rasouli and colleagues have recently published evidence that, compared with healthy controls, CFS/ME patients show poorer gross motor function, assessed by taking longer on a reaction time task, and also poorer fine motor function, assessed using a pegboard task [79].

Increased symptom intensity in CFS has been reported to occur as a result of neuromuscular strain, with such strain (as opposed to sham strain) being associated with increased somatic pain and problems with concentration [80].

A whole-genome sequencing study in 95 CFS/ME patients and 77 age- and sex-matched controls, by Johnston and colleagues, has shown preliminary evidence of a higher prevalence of a SNP in the adrenergic receptor α1 (*ADRA1A*) in the patient group [81]. Further study of adrenergic receptors in CFS/ME/SEID is clearly indicated.

## Calcium Ion Mobilisation

Recent evidence from the work of Nguyen and colleagues points to the importance of natural killer (NK) cell calcium ion mobilisation in CFS/ME/SEID [82]. CD56^bright^ CD16^dim/−^ NK cells are important in immunosurveillance and produce relatively large amounts of cytokines. In contrast, CD56^dim^ CD16^+^ NK cells are cytotoxic, killing infected cells, tumour cells and cells which are ‘missing self’. Compared with healthy controls, immunosurveillance NK cells (of the first type just mentioned) from CFS/ME patients have been found to have reduced transient receptor potential melastatin subfamily 3 ion channels, which are involved in calcium ion signalling [82]. When CFS/ME-patient NK cells of this type were stimulated with pregnenolone sulphate (sulfate), they showed increased calcium ion flux compared with the same NK cell type from the control group [82].

While it is too early to speculate on the implications of the above results, they point to the intriguing possibility that calcium ion mobilisation in NK cells may well be important in the pathogenesis of CFS/ME/SEID. This is consistent with the importance of intracellular calcium ion concentration in Pall’s NO/ONOO^−^ cycle [15, 18], mentioned earlier in this paper. Moreover, Pall has also pointed out the association of CFS/ME/SEID with MCS, with both disorders being associated with increased NMDA activity [18–20, 22]. Furthermore, our group have recently found that desensitisation treatment for chemical and food sensitivities using low-dose immunotherapy ascertained by provocation neutralisation is itself associated with reduced influx of calcium ions into lymphocytes [83]. Thus, it seems reasonable to suggest that this particular therapeutic intervention should be formally studied in a trial in CFS/ME/SEID patients. We now turn to treatment approaches which have already been the subject of published studies.

## Treatment

In this section, published evidence of the efficacy of some biological, non-psychological, non-psychiatric interventions for CFS/ME/SEID are described. (One physician, himself bedridden with ME, has even argued that graded exercise therapy and cognitive behavioural therapy may be harmful to patients [84].)

From the description of the NO/ONOO^−^ cycle earlier in this paper, it follows that downregulation of this cycle might be expected to be of therapeutic value in CFS/ME/SEID. One candidate to achieve such downregulation is vitamin B_12_, which is available over the counter in different forms, including as hydroxocobalamin, cyanocobalamin and methylcobalamin. In 1995, using porcine endothelial cells from the aorta, Rochelle and colleagues published evidence favouring the existence of a redox reaction between reduced cobalamin and NO, with binding of NO occurring in a reversible manner to oxidised cobalamin [85]. Since then, several studies, both in vitro and in vivo, have established the NO scavenger role of cobalamin [15, 86]. This may help explain the results of an early double-blind cross-over trial in patients complaining of tiredness (the study was from the 1970s, well before the publication of more recent operational criteria for the diagnosis of CFS/ME/SEID), in which twice-weekly injections of hydroxocobalamin, for a fortnight, were associated with improved well-being which persisted for at least a month [87]. At the time of writing, there have been no publications of any similar trials in operationally defined CFS/ME/SEID, although anecdotal reports suggest that there may be a benefit of this vitamin in such patients, with this benefit not necessarily being associated with a pre-existing vitamin B_12_ deficiency [15, 88–90].

The lipophilic coenzyme known as coenzyme Q_10_ (CoQ_10_) has important roles in ATP generation, inflammatory cascade inhibition and apoptosis prevention [91]. CoQ_10_ can act as an antioxidant which can inhibit the oxidation of DNA, lipids and proteins [92]. It can also prevent the initiation and propagation of lipid peroxidation, including by means of the recycling action of NAD(P)H:(quinone acceptor) oxidoreductase 1 activity [92–94]. These actions occur in vivo in mammals; murine experiments have shown that dietary supplementation is associated with reduced lipid peroxidation [95, 96]. This occurs also in membranes of mitochondria located both peripherally and in the CNS and is associated with improved mitochondrial function, again both peripherally and centrally [97, 98]. Therefore, it would seem reasonable to propose that supplementation with CoQ_10_ and NADH could be beneficial in CFS/ME/SEID. In a recent double-blind 8-week study by Castro-Marrero and colleagues of 80 CFS patients who were randomised to receive either this supplement combination or a matching placebo, the active group showed a reduced maximum cardiac rate during a cycle ergometer test at the end of the study (compared with baseline) and indeed also a reduced perception of fatigue [99]. It is of interest to note that another mitoprotective dietary intervention which has been proposed for CFS/ME/SEID patients is caloric restriction, but this has not yet been the subject of a published trial in this disease [100].

The mainly pineal neurohormone melatonin (*N*-acetyl-5-methoxytryptamine) has important antioxidant, neuroprotective and immunomodulatory properties and may help prevent or treat bacterial and viral infections [101]. Furthermore, the normal circadian rhythms of fatigue and of urinary melatonin levels covary [102], while administration of this indole to healthy volunteers has been found to be associated with a reduction in self-rated fatigue or tiredness [103]. It seems reasonable, therefore, to suggest that melatonin supplementation may be of therapeutic value in CFS/ME/SEID patients. While some earlier pilot studies in CFS/ME/SEID gave essentially negative results, or, in one case, there was evidence of high nocturnal melatonin levels therefore suggesting that it would be inappropriate to administer melatonin, a few more recent studies have given positive findings [104–107]. On balance, the evidence suggests that it would be appropriate to conduct larger, randomised, double-blind trials of melatonin supplementation in CFS/ME/SEID patients [108]. Meanwhile, it would appear sensible to advise CFS/ME/SEID patients, and indeed most people whether ill or not, to avoid the prolonged evening use of electronic gadgets that employ light-emitting diode screens giving off relatively high levels of blue light (with a wavelength of approximately 470 nm), as exposure of healthy volunteers to such light for just half an hour, starting at 8 p.m., is associated with a strong suppression of nocturnal melatonin production (of over 90%) [109].

The association of CFS/ME/SEID with EBV or HHV-4 infection has been mentioned above. The use of antiviral treatment for infectious mononucleosis is controversial and is not currently routinely recommended [110]. However, in the mid-1990s a small, double-blind, placebo-controlled, phase III cross-over study of antiviral treatment in CFS patients showed promising results, with continued improvement in symptomology with up to 18 months’ treatment [111]. It could therefore be argued that a larger trial in CFS/ME/SEID would be in order. Meanwhile, it may be prudent to consider the role of the intestinal microbiota and virome, with the use of suitable supplementation (although again evidence from clinical trials is currently lacking) [112].

Twenty nine CFS/ME patients took part in an open-label study of the monoclonal anti-CD20 antibody rituximab, administered as a couple of infusions a fortnight apart followed by maintenance treatments [113]. Use of this monoclonal antibody is associated with depletion of B lymphocytes; in this study, clinical improvements were observed in almost two-thirds of the participants, with remission being maintained at the time of 3-year follow-up in a majority of the responders [113]. At the time of writing, it is not yet known how to differentiate responders from non-responders, although it has been noted that responders have lower levels of baseline serum IgG [114]. Furthermore, in late 2017, it was reported that preliminary results from a phase III trial of rituximab in CFS/ME may have been associated with negative findings; the authors feel that it would be prudent to await formal publication of the full results before commenting further.

Unsurprisingly, in light of the above findings, it has recently been suggested that rehabilitation for CFS/ME/SEID patients should be extended from a narrow psychologically based domain and become multidisciplinary, including for example exercise physiologists and physiotherapists [115].

## Conclusions and Future Directions

Strong evidence has been presented which points to a molecular neurobiological aetiology of CFS/ME/SEID. Accordingly, it is suggested that biologically based therapeutic interventions should constitute a focus for future research. As has been seen above, preliminary trial data already point to the efficacy of such an approach.
